# The search for the ultimate exercise countermeasure to preserve crew health and ensure mission success in long‐duration spaceflight

**DOI:** 10.1113/EP091737

**Published:** 2025-02-07

**Authors:** Rodrigo Fernandez‐Gonzalo, Stefan Schneider, Martina Heer, Anna‐Maria Liphardt

**Affiliations:** ^1^ Department of Laboratory Medicine, Division of Clinical Physiology Karolinska Institutet Stockholm Sweden; ^2^ Unit of Clinical Physiology Karolinska University Hospital Stockholm Sweden; ^3^ Center of Health and Integrative Physiology in Space German Sport University Cologne Germany; ^4^ IU International University of Applied Sciences, Health Sciences Erfurt Germany; ^5^ Department of Internal Medicine 3 – Rheumatology & Immunology Universitätsklinikum Erlangen & Friedrich‐Alexander‐Universität Erlangen‐Nürnberg Erlangen Germany; ^6^ Deutsches Zentrum Immuntherapie Universitätsklinikum Erlangen & Friedrich‐Alexander‐Universität Erlangen‐Nürnberg Erlangen Germany

**Keywords:** deep‐space mission, exercise countermeasure, Mars, Moon, spaceflight

## Abstract

The current understanding of crew health maintenance is founded upon decades of physiological research conducted in terrestrial spaceflight analogues and in low Earth orbit, particularly on the International Space Station. However, as we progress towards the Lunar Gateway and interplanetary missions, it is imperative that the tools employed to maintain crew health are redefined, including the utilisation of exercise countermeasures. The successful implementation of exercise countermeasures for deep space missions must address a number of challenges, including those posed by new environments with elevated levels of cosmic radiation and solar particle events, extended mission durations and constrained space availability. In this Topical Review, the authors address points that are important (and sometimes critical), but often ignored, in order to define future exercise countermeasures for long‐duration space missions. Multi‐organ countermeasures, countermeasure enjoyment, time‐dependent load variability, the relationship between nutrition and the success of exercise countermeasures, and the individual variability in response to a given countermeasure are presented and discussed. The aim of this article is to raise awareness of important aspects that can profoundly influence the efficacy of exercise countermeasures, thereby affecting the health of the crew and the success of the mission during prolonged spaceflight.

## Abstract (highlights)

- **What is the topic of this review?**
  This Topical Review aims to highlight the significance of key factors that can profoundly influence the efficacy of exercise countermeasures, and consequently, the crew health and mission success during long‐duration spaceflight. The review places particular emphasis on aspects that are of crucial importance, yet frequently overlooked in this context.
- **What advances does it highlight?**
  Multi‐organ countermeasures, countermeasure enjoyment, time‐dependent load variability, the relationship between nutrition and the success of exercise countermeasures, and the individual variability in response to a given countermeasure in the context of lunar and deep‐space missions are presented and discussed.

## 1 INTRODUCTION

For thousands of years, humans have been attracted to and fascinated by the Moon, the stars and what may lie beyond. We have tried to understand these phenomena through a range of methods, from mere observation to active exploration. We are fortunate to live in one of the most exciting periods of human space exploration, where travel into deep space for long periods of time is becoming a tangible reality. One of the pivotal considerations in attaining this objective is ensuring crew health throughout the mission, which is indispensable for mission success. With long‐duration, exploratory space missions on the horizon and the health of the crew at stake, it is not surprising that space agencies are allocating a considerable amount of resources to the development of countermeasures designed to offset or mitigate the anticipated health and performance‐related challenges associated with forthcoming missions beyond low‐Earth orbit. A fundamental element of these countermeasures is the utilisation of exercise training devices and protocols.

In this Topical Review, we approach the question of defining future exercise countermeasures for long‐duration space missions, emphasising points that, while being important (and sometimes critical), are often ignored. Multi‐organ countermeasures, countermeasure enjoyment, time‐dependent load variability, the relationship between diet and the success of exercise countermeasures, and the individual variability in response to a given countermeasure are presented and discussed. Our aim is to raise awareness of important aspects that can have a significant impact on the success of exercise countermeasures, and thus on crew health and mission success during long‐duration spaceflight.

### 1.1 Human spaceflight and health challenges

Over millions of years, the human body has evolved to meet the physical demands on Earth and has developed physiological strategies to adapt to the Earth's gravitational pull. For example, venous valves and the peripheral vasculature allow deoxygenated blood to be effectively returned to the heart. The vertebral column and the muscles that support this structure can resist gravity and allow for an upright posture, which has been evolutionarily advantageous. More than 60 years ago Yuri Gargarin started the human space exploration adventure becoming the first person ‘outside’ the Earth and exposing the human body to this hostile environment for the first time. From the very beginning, we were aware that the space environment is a challenge to human health (Kitzes, 1959). Today, there is agreement that the ‘space exposome’ induces physiological changes to the human body that are detrimental to health and performance (Crucian et al., 2018; Demontis et al., 2017) during the mission but also upon return to Earth. Microgravity and radiation were early considered and tried to be accounted for, together with stress related factors and confinement issues. For example, if no countermeasure of any type is employed, the skeletal muscle mass decreases by 4–10% in 8 days under microgravity conditions, alongside even further decrements in muscle force and power production (LeBlanc et al., 1995; Narici et al., 2003). Our bones become frail, our joints get weaker, our maximal aerobic capacity, an indication of our endurance, worsens at an alarming speed and our immune system is dysregulated. These are only some examples, but basically all the physiological systems and organs of our body suffer the consequences of the space exposome (Krittanawong et al., 2023). After return from Space, crew members participate in rehabilitations programmes to support the re‐adaptation of the human body to gravity conditions. While some reports have been published (Hides et al., 2021; Payne et al., 2007; Petersen et al., 2017), post‐flight recondition regimens are mostly not researched in a standardized way, and experiments often do not comprise long term follow up visits. More recent investigations of longer‐term recovery of bone tissue reveal that the level of recovery reached is highly individual and often incomplete (Burkhart et al., 2020; Gabel et al., 2022; Vico et al., 2017; Walle et al., 2024). This underlines the need for more systematic research during the post‐flight period (Liphardt et al., 2023).

The nature of the stressors that affect the human body in space is key to developing effective countermeasures. As mentioned above, (i) microgravity, (ii) radiation, and (iii) stress and confinement are generally described as the three factors that may pose negative health consequences. Each of these stressors affects the body organ systems differently, and they can interact with each other, amplifying the magnitude of the consequences on human health (Radstake et al., 2023). It is then important that future countermeasures consider the variety of the components of the space exposome. On Earth, a good example of a ‘multi‐stressor countermeasure’ that alleviates the effects of inactivity (lack of muscle action similar to microgravity) and radiation in patients is physical exercise (Alkner & Tesch, 2004; Lipsett et al., 2017). As exercise has been shown to decrease stress levels as well (Kettunen et al., 2015), it is clear that any countermeasure protocol for long‐duration spaceflight will include exercise routines. What exercise type(s) and how much exercise is needed or recommended, and how exercise interacts with other potential countermeasures are key questions that need to be answered as soon as possible to foster deep‐space exploration.

Our current knowledge about maintaining crew health is based on decades of physiological research in Earth‐bound spaceflight analogues and in low‐Earth orbit, primarily on the International Space Station (ISS). However, as we move towards a Lunar Gateway and interplanetary missions, the tools used to maintain crew health need to be redefined, including exercise countermeasures, and maybe also the view on what needs to be achieved in terms of physical function of the crew. Indeed, successful exercise countermeasures for deep space missions will need to be designed considering aspects like longer mission durations, less available space and higher exposure to cosmic radiation and solar particle events.

## 2 THE ELEPHANT IN THE (SPACE) ROOM—MISSING POINTS WE ARE ALL AWARE ABOUT EXERCISE COUNTERMEASURES, BUT WE SEEM TO DOWNPLAY OR IGNORE

It is clear that exercise is and will remain an important part of the countermeasure strategy for human deep space exploration. While there are continuous efforts to create new exercise devices and protocols, there is a great potential to improve exercise countermeasure success by broadening the research focus to other aspects that are critical to the success of exercise countermeasures but do not necessarily involve device or protocol development. While these aspects—which we highlight in the following paragraphs—may be difficult to address scientifically and systematically, they may become crucial for the success of any exercise regimen for interplanetary space travel.

### 2.1 Multi‐organ countermeasure

The room available for both exercise devices and crew time is very limited during current space missions. Space available for exercising devices will be even more limited for Lunar Gateway (at least during the first years) and deep space missions. However, the crew time available for exercise sessions may increase. In any case, considering the constraints in the Orion multi‐purpose crew vehicle (Laws et al., 2020), and at least for the initial Lunar Gateway missions and future deep space missions, exercise countermeasures should follow the principle of ‘one (or as few as possible) size fits all’. Exercise has global effects on the human body and both aerobic and resistance exercise training, when used wisely, can have effects on the musculoskeletal, cardiorespiratory and immune systems (Fernandez‐Gonzalo et al., 2012; Lundberg et al., 2013; Schjerve et al., 2008), for example. However, some exercise routines are better suited to different organ systems than others (e.g., resistance exercise has greater effects on muscle size and function than on cardiac parameters). It is also important to consider potential negative consequences of specific exercise countermeasure or protocols targeting one organ in other systems/organs. This could be the case, for example, if the joints are weakened during spaceflight (Lambertz et al., 2001) and the training loads are too high, which could lead to pain, inflammation and serious injuries.

Although most researchers are aware of the global effects of exercise, only recently have research groups with different focuses (e.g., muscle vs. cardiorespiratory vs. brain vs. immune system) begun to work together to truly understand the multi‐organ effects of the countermeasures employed (Scott et al., 2023). Space agencies play an important role here and should encourage collaboration between research groups with different expertise by prioritising multi‐organ projects. Researchers should also understand and encourage these multidisciplinary collaborations. The data from such studies could be analysed using modern biostatistical approaches to understand the role of each organ and the interactions between different organs in the adaptations induced by the space environment and the exercise countermeasures employed. Once this information is available, it will be easier to select a specific exercise routine that can truly serve as a multi‐organ countermeasure during space missions.

### 2.2 Enjoyment of countermeasure routines

When preparing athletes for international competition, whatever the discipline, the aim is to maximise performance. This is no longer just a physiological process, but let us leave aside the mental aspects for a moment. Total performance maximisation is first and foremost about the demands of the sport. High jumping, for example, requires explosive jumping power. And a clean run‐up technique. If the last stride of the run‐up is long, you need a softer muscle–tendon complex that can store energy. If the last stride is short, the muscle–tendon complex must be rather hard. If an athlete has a long final stride combined with a hard muscle–tendon complex, the training implications are either to change the technique or to ensure that the muscle–tendon complex becomes softer through appropriate training stimuli (Kubo et al., 1999)—a very sophisticated approach.

Most people who exercise today are not primarily concerned with maximising performance. Most people want to improve their health (Ambrose & Golightly, 2015; Deslandes et al., 2009; Smith & Merwin, 2021). However, the training and fitness advice they receive is usually based on the philosophy of the person making the advice (and, of course, the money they make from it), rather than the wishes or needs of the person wanting to improve their health.

When preparing people for a flight to the Moon or Mars and a prolonged stay under the space exposome, it would seem that it is not important to identify training recommendations to maximise performance, but rather to optimise performance. All too often, however, relevant questions from exercise science are transferred to astronaut training without any previous reflection. The question of what is best for the individual systems (bone/muscle/cardiovascular/cartilage) may be relevant from a scientific perspective—and certainly interesting to observe. But regarding human performance, however, the question arises: does it make sense?

Improving or maintaining performance is only possible through regular exercise. We all know how many people find it difficult to get up and go to the gym on a regular basis. The key to improving performance is less about the type of training and more about the motivation to train regularly (Garber et al., 2011). This means that training to improve and maintain performance is primarily a matter of the mind. This is the biggest challenge, particularly in the health sector: finding the motivation to exercise regularly and at the right intensity to produce physiological adaptations. This is where modern training methods come in, focusing not on maximising physiological performance, but on motivation and enjoyment of exercise (Chakraverty et al., 2024) to ensure that the individual is exercising independently of the frequency, intensity or type of exercise (at least if the WHO recommendations are met!).

A similar scenario should be found for future long‐term missions (European Space Agency, 2022). When discussing issues related to their daily exercise regimen, current crew members seem to have an intrinsic motivation to exercise. This motivation is partly related to the physical challenges of a long duration mission, but is also a result of their lifestyle. We find significantly higher intrinsic motivation to exercise among well‐educated people (Dennis et al., 2022; Stalsberg & Pedersen, 2010; Tandon et al., 2021). This certainly has a lot to do with socialisation and access to sport and exercise opportunities, leading to sport and exercise being seen as part of the self‐image. And exercise routines are simply part of their lifestyle.

Moreover, there are also differences in preferences that need to be considered. For example, some individuals prefer strength and conditioning over aerobic exercise or training routines. Some prefer short bouts of exercise with high intensities, others prefer continuous longer lasting exercise.

Training plans should therefore be backed up by scientific data, but in close consultation with the people who exercise. Especially if these people are very passionate about their bodies and exercise. The other way around would be even better: people who want to exercise should tell us what they like to do, how they like to do it, what their goals and motivations are—and science should take this as a guideline and create optimised training plans.

In summary, to counteract the negative effects of the space exposome and maintain the crew's physical fitness while regulating their stress to maintain their cognitive flexibility and emotional well‐being, we need (i) motivation and commitment from the trainee, (ii) individualised training plans based on the trainee's wishes and preferences, and (iii) communication between all parties.

### 2.3 Load variability and load control, compliance and monitoring

The load during exercise countermeasures is typically controlled to adjust the training loads so that an anabolic stimulus is achieved. With long‐term exposure to the space exposome, it will be particularly important to better understand the resulting forces in the body during exercise and occupational tasks. Such knowledge must be integrated into the framework of musculoskeletal tissue adaptation. Exercise and occupational loads need to be controlled considering musculoskeletal tissue deterioration and nutritional requirements (see below). Musculoskeletal tissues will atrophy during spaceflight. We have a rough idea of what this deconditioning looks like for certain tissues, but only for short‐term missions. There is a dearth of data pertaining to missions exceeding 6 months in duration. In addition, complex structures such as joints remain insufficiently researched. The functionality of joints is contingent upon the intricate interplay of connective tissue, muscles, bones and cartilage. The failure of any single component carries a significant risk of injury. To gain a deeper understanding of the actual and ideal load variations and to identify strategies for preventing failure, a multidisciplinary research approach is essential.

Obtaining the aforementioned information would lead to personalized load management. This would consequently require the implementation of in‐flight measures that allow load control. Biomechanical modelling approaches would prove beneficial in understanding the change of mechanical properties in the relevant tissues, thereby facilitating load control throughout the mission with the aim to avoid failure of the musculoskeletal system (e.g., ligament rupture, cartilage defect). While first studies in this context are conducted (Bärligea et al., 2024; Poveda et al., 2024), it is essential to ensure that the kinematic models employed are carefully designed to avoid oversimplification and the inadvertent overlooking of potential risks. To achieve this and to allow monitoring of tissue adaptation during flight, different monitoring tools need to be developed. These should enable (i) monitoring of movement (e.g., based on wearable sensor motion capture; Ravizza et al., 2023) and (ii) monitoring of musculoskeletal tissue status (based on simple imaging tools like ultrasound or other imaging techniques, and/or biomarkers of musculoskeletal tissue turnover), especially for those tissues where data for space adaptation are not yet available. Subsequently, the data sets can be incorporated into models for load estimation.

Space agencies are trying to coordinate human life science research by identifying research gaps that are often one‐dimensional—possible causalities should be identified and interactions better explored. As previously outlined, inadequate load management of the musculoskeletal system can precipitate joint injuries. This risk becomes more likely with increasing mission duration. Joints are complex organs comprising different tissues with very different recovery capabilities. Joint injuries are complex, and treatment in space is very difficult. Failure of the joints could result in the inability to perform any exercise countermeasure, which would trigger a chain reaction that increases other risks.

There is considerable variation in the extent to which individual crew members adhere to the prescribed exercise regimen, with the regularly referenced 2 h of exercise per day (Fraser et al., 2012; Hughson et al., 2016) not being consistently met. This is because the exercise time encompasses activities such as the preparation of the exercise equipment and personal hygiene routines. To address these issues, the pre‐flight period can be utilized to gain a deeper understanding of each crew member's exercise behaviour, intrinsic motivation (as previously discussed), self‐regulation and realistic expectations. These factors are crucial for ensuring optimal adherence to any exercise training programme. Furthermore, it is vital to ensure that the significance of adhering to exercise countermeasures is explicitly conveyed to the crew. This should include an explanation of the potential consequences for personal health and mission success if exercise routines are not followed, particularly in the context of deep space missions where exercise programmes will be a fundamental requirement. The implementation of an effective educational programme in this field would serve to enhance the level of compliance of the crew to the exercise countermeasures in question, whilst simultaneously facilitating their ability to devise and implement such programmes in a manner that is both enjoyable and conducive to their personal wellbeing.

An important aspect related to the exercise load during space missions is the concept of the minimal effective dose of exercise required. After all, astronauts are not athletes who ‘have to’ increase their maximum physical performance, but rather they need to maintain their physical ability to ensure the success of the mission and avoid future, long‐term negative health effects of spaceflight. While a limited number of studies, like the European Space Agency's MNX (Medium duration nutrition and vibration exercise) and STBR‐AG2 (Short‐Term Bed Rest Artificial Gravity 2) bed rest studies, have attempted to address this question by employing countermeasures with very low intensity and volume (Liphardt et al., 2024, 2020), this line of research is still largely unknown. This is somewhat surprising as it could help fine‐tune exercise countermeasure and time management during missions. Some reports suggest that this minimal effective dose is not high for some physiological systems, for example, skeletal muscle (Tesch et al., 2004), although there is currently no integrative study analysing this for multiple organ systems. However, one factor to consider is that the ‘exercise time’ is also used by astronauts to unwind and relax, and reducing this time to a minimum, while serving to maintain bodily functions, may be detrimental to the individual's wellbeing and potentially to the success of the mission. It is therefore clear that studies that bring together the different views on the minimal effective dose of exercise during space missions are needed.

### 2.4 Relationship between exercise countermeasure and diet

While the relationship between diet and exercise success is well understood for athletes and the general population, it is crucial to emphasize that in the context of spaceflight, proper diet plays an even more significant role in optimizing exercise outcomes. Space missions have been and to some extent still are characterized by insufficient energy intake leading to loss of body mass, with many astronauts consuming only around 70% of their predicted needs during Apollo to Shuttle missions, and around 80% on the ISS (Smith et al., 2021). The only exception is space travellers who participated in metabolic studies that comprised following a balanced, controlled diet during Skylab missions (Leach & Rambaut, 1977; Rambaut et al., 1977), European flights to Mir (Drummer et al., 2000) and ISS missions (Smith et al., 2012). Reduced energy intake results in loss of lean mass (1–15% (Smith et al., 1999, 2005, 2012, 2014)) and bone tissue (Baek et al., 2008; Ihle & Loucks, 2004), and the available spaceflight data suggest an average body mass loss of 2.4% per 100 days in space (Laurens et al., 2019).

Lower calorie intake leads to a significant loss of body mass on the ground (Brozek et al., 1957) (Faintuch et al., 2000) and during Apollo missions (Smith et al., 2021). Inadequate dietary energy intake can lead to severe muscle atrophy and tissue breakdown, especially when exercising (Saenz et al., 2024), and in extreme cases result in permanent organ damage and even death (Stratton et al., 2003). Adequate caloric intake is critical, as insufficient consumption not only leads to loss of adipose tissue, but also decreases protein synthesis during spaceflight (Stein et al., 1999) and increases protein breakdown (Biolo et al., 2007) to utilize amino acids as an energy source. Semi‐starvation affects the cardiovascular system (reviewed in Smith et al., 2021), for example by reducing orthostatic tolerance, and initiates inflammation (Bosutti et al., 2008; Heer et al., 2019). Indeed, a bed rest crossover study evaluating the impact of hypocaloric nutrition on multiple systems found that undernutrition exacerbated the negative effects of bed rest on human physiology (Biolo et al., 2007). This study also showed that hypocaloric nutrition was linked to lower systolic blood pressure and heart rate responses during lower body negative pressure application, indicating reduced cardiovascular performance (i.e., orthostatic tolerance) (Florian et al., 2015). Insufficient caloric intake could also impair motor and cognitive functions, which will impact the performance of space travellers during essential work‐related tasks during landing (O'Leary et al., 2024). Research in the military, where negative energy balance is common during operations, has established a link between overall energy balance and lower body strength (Murphy et al., 2018). Therefore, astronauts experiencing semi‐starvation may first show a decrease in endurance and then a decrease in muscle strength, which is associated with a loss of muscle mass.

As described above, maintaining physical function is important for mission success and especially during long‐duration and interplanetary missions, resistance and endurance exercise training will be implemented to preserve musculoskeletal tissues, prevent loss of lean body mass and maintain cardiovascular health and glucose metabolism (Booth et al., 2012). However, adequate energy intake (balanced diet with adequate energy and protein content) is critical to support the success of exercise routines and prevent the muscle breakdown that can occur with inadequate dietary energy intake.

Astronaut diets are often high in protein (Smith et al., 2021). Adequate dietary intake of energy, protein and calcium, combined with strength training, has been shown to maintain bone mineral density in spaceflight (Smith et al., 2012). Considering that dietary protein content is the primary factor determining the potential renal acid load of a diet (Remer & Manz, 1994), a lack of enough base precursor‐containing foods like fruits and vegetables could result in the generation of an acid‐retaining state with high‐protein diets. This would in turn prompt the skeletal acid–base homeostatic systems to release alkaline salts to maintain acid–base balance (Wesson, 2021). Changes in acid–base balance can affect muscle protein metabolism, which may result in a gradual decrease of lean body mass over time (Moore et al., 2017). Conversely, acute exercise and protein intake independently and synergistically activate anabolic pathways, helping to maintain a neutral or positive muscle protein balance during spaceflight (Hackney & English, 2014). However, even moderate reductions in blood pH can lead to bone resorption and a progressive decrease in bone mineral content. Increased bone resorption has been observed during bed rest, where high protein intake resulted in elevated bone resorption markers (Heer et al., 2017; Zwart et al., 2005). In spaceflight, increase of base precursors through higher fruit and vegetable intake may protect against bone mineral content loss when adequate calcium is consumed, especially if no resistive exercise is performed (Zwart et al., 2018).

Total energy expenditure comprises various energy costs: resting metabolic rate (essential processes for life), physical activities, digestion, absorption and metabolism of food (diet‐induced thermogenesis) (Westerterp, 2013). The energy consumed during different activities varies depending on the type of activity, its duration and intensity. For example, resistance training generally consumes less energy compared to endurance training as shown in studies by João et al. (2021) and Meijer et al. (1991). A comparison of energy needs in resistance and endurance exercise as described in these studies demonstrates that endurance exercise requires higher energy intake to balance out the energy consumption.

On deep space missions, it is not possible to transport all the food that will be required; procedures are needed to produce enough calories and essential nutrients (e.g., macronutrients, vitamins, minerals and antioxidants) during the flight and on other planets. The development of food production and cultivation methods suitable for extra‐terrestrial environments is critical and calculations show that the number of exercise sessions is directly related to the energy requirements. Therefore, it is essential to find a balance between the exercise volume required for preserving physical function and the limitation of food storage and production. The question of the optimal exercise regime to maintain health during interplanetary missions and the amount of food required to compensate for the energy expended is a delicate equilibrium that must be managed effectively. Immediate research is urgently needed to explore the optimal balance between dietary and exercise regimes during space and interplanetary missions.

### 2.5 Individual variability

The fact that two people can respond differently to the same physiological stimulus is well known and has been demonstrated in clinical trials, for example, with drugs and exercise interventions (Ross et al., 2019; Smith & Rawlins, 2013). This is also the case with astronauts when they perform the exercise countermeasures on board the ISS, as indicated, for example, in the performance and phenotypic adaptations of astronauts after an in‐flight high intensity training programme (English et al., 2020), or in molecular adaptations to the classic exercise routines on the ISS (Blottner et al., 2023). Thus, we can now envision a scenario where space agencies are able to determine which of their astronaut candidates responds better to the various exercise countermeasures. This would undoubtedly help to ensure the health of the selected crew members and thus the success of the mission. Unfortunately, this is not as simple as it may sound. By studying individual variability in response to an intervention, researchers (or space agencies) are trying to identify ‘non‐responders’ and ‘responders’ (Mann et al., 2014). Once this is established, the mechanisms influencing the differential response (i.e. moderators of the individual response) can be identified, with the ultimate goal of providing individualized and personalized interventions (Buford et al., 2013). It is clear that an important step in this process is to accurately determine actual biological individual variability as opposed to technical and random variability (Atkinson & Batterham, 2015; Atkinson et al., 2019). Previous research on this topic has sometimes ignored confounding factors (technical and random variability) that prevent the determination of the true biological variability to the physiological intervention in question.

What strategies can space physiologists use to determine the actual individual biological variability? This is a question that should be carefully addressed by space agencies and researchers if they are truly interested in understanding individual variability in response to a particular exercise countermeasure. It is important that this issue is considered before the study is designed.

Apart from using well‐defined and appropriate statistical methods (described in detail in e.g., Hecksteden et al., 2015 and Atkinson & Batterham, 2015), one of the most important aspects is a robust control group (Atkinson & Batterham, 2015; Hopkins, 2015). This is because the inter‐individual response of prospective astronauts to an exercise countermeasure can only be determined if we know the random and technical variability in a similar group of participants under comparable environmental conditions (age, sex, physical condition, season, diet, etc.). The more similar the study groups are and the more controlled the situation is between pre‐ and post‐test, the better the model is suited to determine the biological variability to the particular exercise countermeasure. It is also important that the groups are sufficiently powered (i.e., sufficient number of observations) so that the range of responses to the intervention recorded is relevant. One way to implement this in crewed spaceflight countermeasure research is to have a control group on the ground that represents each astronaut crew in space, as previously proposed (Liphardt et al., 2023).

However, it may prove difficult to gain access to a control group that meets the characteristics described above. An alternative approach is the utilisation of cross‐over designs, wherein the same individual participates in both the intervention and control phases, separated by a wash‐out period. This model reduces the number of participants required, and genetic factors and past physical activity, for example, can be controlled for. Nevertheless, the use of cross‐over designs presents certain challenges in terms of controlling for external factors, such as the time of year or age (if the intervention and wash‐out phases are lengthy). In any case, a study demonstrated considerable and reproducible individual variability in muscle mass and strength following bed rest, which is considered the gold standard for spaceflight analogues (Fernandez‐Gonzalo et al., 2021). This evidence supports the efficacy of the cross‐over design approach.

Another aspect that can help to isolate and eliminate any contamination from random and technical variability is to perform multiple samplings per time point, if possible, or at least multiple samplings in the baseline condition. The latter is a straightforward and simple approach that should be implemented by space agencies in the period between crew definition and launch to identify and remove random and technical variability from future analyses.

Most of the research on individual variability in human spaceflight is devoted to the discovery of biomarkers that would define a high‐responder or low‐responder (Schmidt & Goodwin, 2013). Many of the potential biomarkers proposed are based on biological samples. In these cases, it is very important that all samples are run and analysed simultaneously, as the batch‐to‐batch effect, for example in omics analyses, can be considerable and strongly influences the results, and may conceal subtle changes that may still be physiologically relevant. This approach also limits potential differences in protocols that may exist when samples are processed within months (or sometimes years) in between (e.g., batch of buffers or kits used, laboratory equipment or the person performing the analysis).

We now invite the reader to imagine the best‐case scenario in which researchers are finally able to determine the individual variability in physiological responses to an exercise countermeasure, identify the moderators of that variability and manipulate those moderators to optimize the countermeasure for a particular individual. It looks like it is done, right? Well, not quite. In fact, we would only have obtained less than a quarter of the information needed to develop truly personalized exercise countermeasures for space travellers. The astronauts carrying out the exercise countermeasure will be exposed to the space stressors: microgravity, radiation and stress and confinement. Each of these individual stressors triggers individual responses at an individual level (Bosi & Olivieri, 1989; Ebner & Singewald, 2017; Fernandez‐Gonzalo et al., 2021). Therefore, we need information on the individual variability of the physiological responses to the countermeasure, radiation, microgravity, and stress and confinement. And most importantly, we need to understand how all these factors interact with each other, which may lead to new individual variability responses in exercise countermeasures under space exposome conditions. Are you ready to take on this challenge?

## 3 POSSIBLE SOLUTIONS FOR COUNTERMEASURES DURING LONG‐TERM SPACEFLIGHT

As the distance between the crew and Earth increases, the likelihood of mission control monitoring the crew's training activities decreases. This necessitates the implementation of innovative and effective systems for the provision of real‐time feedback and the control of appropriate exercise techniques and protocol compliance. It is essential that such protocols are planned in accordance with the energy requirements for their execution, given the limitations in food transportation and production capacity during long‐term spaceflight. Accordingly, all activities performed during the flight, especially those that can be considered of a certain intensity, should be included in the calculations of the total ‘training load’. Furthermore, exercise protocols must be tailored to individual crew members in order to enhance the positive effects on multiple physiological systems. Given the lack of information on musculoskeletal adaptations to a long (more than 6 months) space flight, special attention should be paid to controlling the risk of injury and any critical level of deconditioning, which must be established in advance. To ensure optimal compliance with any proposed exercise regimen, it is essential to enhance the enjoyment of the activities involved. Ultimately, the most effective exercise is the one that is performed. And exercise is most likely to be undertaken when it is fun.

## AUTHOR CONTRIBUTIONS

All the authors participated in manuscript drafting and review. R.F.‐G. coordinated the process. All authors have read and approved the final version of this manuscript and agree to be accountable for all aspects of the work in ensuring that questions related to the accuracy or integrity of any part of the work are appropriately investigated and resolved. All persons designated as authors qualify for authorship, and all those who qualify for authorship are listed.

## CONFLICT OF INTEREST

None declared.
